# Glyco-engineering for biopharmaceutical production in moss bioreactors

**DOI:** 10.3389/fpls.2014.00346

**Published:** 2014-07-09

**Authors:** Eva L. Decker, Juliana Parsons, Ralf Reski

**Affiliations:** ^1^Department of Plant Biotechnology, Faculty of Biology, University of FreiburgFreiburg, Germany; ^2^BIOSS Centre for Biological Signalling StudiesFreiburg, Germany; ^3^Freiburg Institute for Advanced StudiesFreiburg, Germany

**Keywords:** *Physcomitrella patens*, moss bioreactor, plant-made pharmaceuticals, glycosylation, posttranslational modifications

## Abstract

The production of recombinant biopharmaceuticals (pharmaceutical proteins) is a strongly growing area in the pharmaceutical industry. While most products to date are produced in mammalian cell cultures, namely Chinese hamster ovary cells, plant-based production systems gained increasing acceptance over the last years. Different plant systems have been established which are suitable for standardization and precise control of cultivation conditions, thus meeting the criteria for pharmaceutical production. The majority of biopharmaceuticals comprise glycoproteins. Therefore, differences in protein glycosylation between humans and plants have to be taken into account and plant-specific glycosylation has to be eliminated to avoid adverse effects on quality, safety, and efficacy of the products. The basal land plant *Physcomitrella patens* (moss) has been employed for the recombinant production of high-value therapeutic target proteins (e.g., Vascular Endothelial Growth Factor, Complement Factor H, monoclonal antibodies, Erythropoietin). Being genetically excellently characterized and exceptionally amenable for precise gene targeting via homologous recombination, essential steps for the optimization of moss as a bioreactor for the production of recombinant proteins have been undertaken. Here, we discuss the glyco-engineering approaches to avoid non-human N- and O-glycosylation on target proteins produced in moss bioreactors.

## INTRODUCTION

Biopharmaceuticals are indispensable in modern medicine. In 2010 more than 200 biopharmaceuticals were available on the market and around 10–20 more are approved every year (Walsh, 2010a). As the biggest group of biopharmaceuticals consists of pharmaceutical recombinant proteins, this term is often used as a synonym for the former. The biochemical and pharmacological properties of a protein are not only determined by its amino acid sequence but also largely influenced by a palette of modifications that proteins undergo co- or posttranslationally (Mann and Jensen, 2003), usually grouped together and referred to as posttranslational modifications (PTMs). Common PTMs found in pharmaceutical proteins are glycosylation, hydroxylation, carboxylation, amidation, sulfatation, disulfide bond formation, and proteolytic processing (Walsh and Jefferis, 2006). Among these, glycosylation is the most frequent PTM, being present in at least 40% of the pharmaceutical recombinant proteins available on the market (Walsh, 2010b). The presence and quality of glycosylation plays a crucial role for the pharmacological properties of a therapeutic protein by influencing protein folding and stability, serum half-life, *in vivo* activity, pharmacokinetics, and immunogenicity (Li and d’Anjou, 2009). Approximately 50% of all eukaryotic proteins are predicted to be glycosylated and this proportion increases substantially with respect to human serum proteins, which are main targets as biopharmaceuticals (Apweiler et al., 1999).

The workhorse for the production of simple proteins is *Escherichia coli*, the best characterized expression system offering high product yields at low costs (Walsh, 2010a). However, this microorganism is not able to perform some PTMs, which are indispensable for recombinant therapeutical proteins (Kamionka, 2011). Consequently, mammalian cell lines are the preferred expression systems for the production of recombinant glycoproteins, as their protein glycosylation patterns largely resemble those of humans (Schmidt, 2004). Among the mammalian cell lines, Chinese hamster ovary (CHO) cells comprise the leading host system for current biopharmaceuticals, even though several CHO-derived products presented non-human glycosylation (Chung et al., 2008; Hossler et al., 2009; Omasa et al., 2010; Kim et al., 2012).

As higher eukaryotes, plants are able to synthesize complex multimeric proteins and perform PTMs in a similar manner as humans do. Therefore, plants and plant cell cultures are gradually gaining acceptance as production hosts for recombinant biopharmaceuticals. The first plant-made pharmaceutical (PMP) received market approval in 2012 () and several additional PMPs are being tested in clinical trials (reviewed in Paul and Ma, 2011). The host system for Elelyso^TM^, a recombinant glucocerebrosidase for the treatment of the lysosomal storage disease Morbus Gaucher, is a carrot-based cell line established by Protalix (Shaaltiel et al., 2007). It is cultured in bioreactors based on disposable plastic bags. While other frequently used plant systems like alfalfa, tobacco, and *Nicotiana benthamiana* need to be grown in greenhouses, bioreactor cultivation is established for the aquatic plant *Lemna minor* and for the moss *Physcomitrella patens* (Decker and Reski, 2007; Paul and Ma, 2011; Paul et al., 2013). Within the following sections we will focus on the special features for biopharmaceutical production and achievements within glyco-engineering of the moss system.

## MOSS CULTIVATION AND ENGINEERING CHARACTERISTICS

**FIGURE 1:**
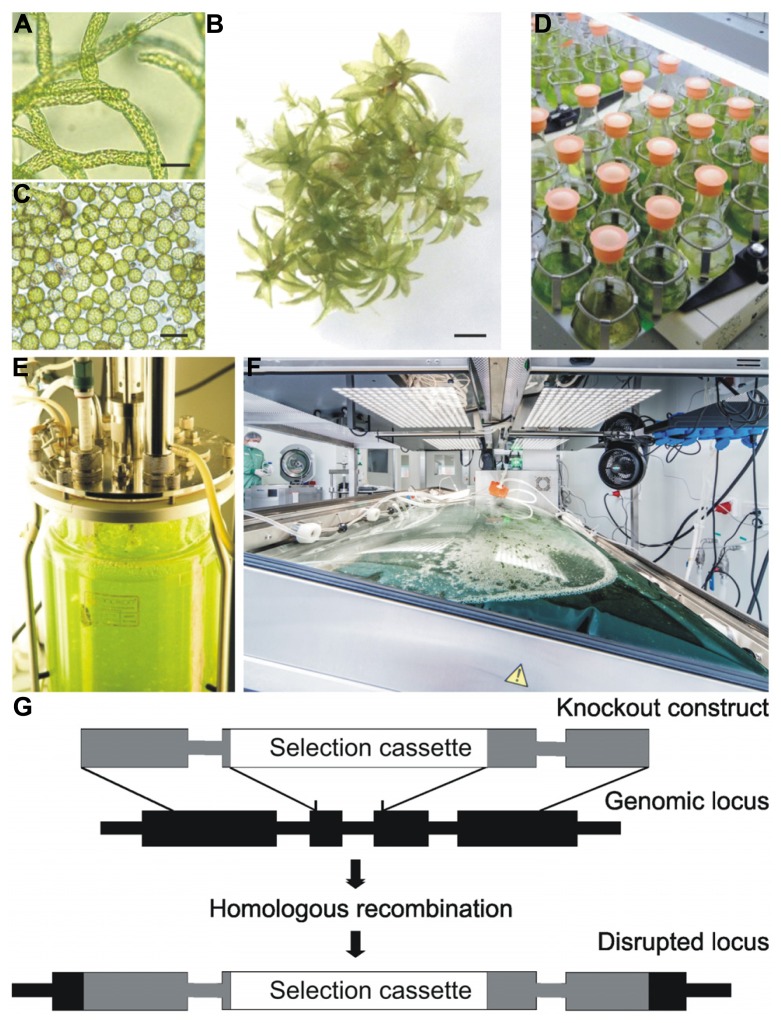
***Physcomitrella patens in vitro* cultivation and schematic representation of a knockout construct for gene targeting. (A)** young filamentous tissue, protonema, ideal for suspension cultures; **(B)** adult leafy moss plant (gametophore); **(C)** protoplasts; **(D)** small scale liquid culture in flasks; **(E)** stirred tank bioreactor; **(F)** wave bioreactor (image courtesy of greenovation Biotech GmbH); **(G)** illustration of allele replacement via homologous recombination. The regions homologous to the targeted gene, which are used for the knockout construct, are shown in gray, and the inserted selection cassette is depicted in white. Thick lines represent introns and rectangles exons. Scale bars 50 μm **(A,B)**, 500 μm **(C)**.

The non-seed plant *P. patens*, a moss, is a well-established model system for evolutionary and functional genomics approaches (Cove et al., 2006; Menand et al., 2007; Mosquna et al., 2009; Khraiwesh et al., 2010; Sakakibara et al., 2013). It can be grown throughout its complete life cycle under contained conditions *in vitro* in a simple mineral medium (Frank et al., 2005; Strotbek et al., 2013). The germination of the haploid spores leads to the growth of protonema (**Figure 1A**), a branched filamentous tissue which comprises two distinct cell types, chloronema and caulonema. Every cell is in direct contact with the culture medium, allowing efficient nutrient uptake and product secretion (Schillberg et al., 2013). This young tissue can be maintained in suspension cultures without any addition of phytohormones, only by mechanical disruption of the filaments. In contrast to immortalized or de-differentiated mammalian or higher-plant cell cultures, which are prone to instability or somaclonal variation (Larkin and Scowcroft, 1981; Xu et al., 2011; Bailey et al., 2012), the fully differentiated protonema tissue is genetically stable (Reutter and Reski, 1996). In the next developmental step, buds differentiating from protonema cells give rise to the adult plant, the leafy gametophore, consisting of shoot-like, leaf-like, and root-like tissues (**Figure 1B**). After fertilization of the gametes, the sporophyte, the only diploid tissue in the life cycle of mosses, grows on and is sustained by the gametophore (Reski et al., 1998). *In vitro* cultivation of all stages can be performed either on agar plates or as suspension cultures in liquid media. The availability of efficient protocols for protoplast isolation (**Figure 1C**) and transfection (Rother et al., 1994; Strotbek et al., 2013) and an excellent regeneration capacity of single transfected cells to whole plants make genetic engineering of moss a straight-forward and frequently used approach (e.g., Lorenz et al., 2003; Qudeimat et al., 2008; Ludwig-Müller et al., 2009; Mosquna et al., 2009; Khraiwesh et al., 2010; Sakakibara et al., 2013). The created moss strains can be preserved by cryo-conservation (Schulte and Reski, 2004), and thus can serve as Master Cell Banks. The International Moss Stock Center IMSC, a reference center for moss ecotypes and transgenic lines, provides a service for long-term storage ().

The employment of *in vitro* axenic plant cell or tissue cultures offers an environment in which contamination with human pathogens is rather unlikely (Schillberg et al., 2013). Moreover, only in these systems culture conditions can be precisely controlled and standardized (Hohe and Reski, 2005), which is essential for the production of pharmaceuticals according to good manufacturing practice (GMP) guidelines (Fischer et al., 2012).

Various scales of highly controllable cultivation devices were developed for *Physcomitrella,* ranging from simple shaking flasks (**Figure 1D**) and 5 L aerated flasks to diverse forms of photobioreactors, including stirred glass tank bioreactors with a volume of up to 15 L (**Figure 1E**; Hohe and Reski, 2002) and a modular tubular bioreactor with a working volume of up to 100 L (reviewed in Decker and Reski, 2008, 2012). More recently, disposable wave-bag reactors (**Figure 1F**) were employed for high-density protein production purposes under full cGMP compliance (). Several pharmaceutically interesting proteins have been synthesized in moss bioreactors, among them the growth factors vascular endothelial growth factor (VEGF; Baur et al., 2005) and erythropoietin (EPO; Weise et al., 2007) as well as the first marketed product for research use, human FGF7/keratinocyte growth factor (KGF; www.greenovation.com). In addition, proteins with a function in immune responses like IgGs (Schuster et al., 2007; Kircheis et al., 2012) and the complement-regulatory protein factor H (Büttner-Mainik et al., 2011) were produced in moss. Furthermore, two products for enzyme-replacement therapies, human alpha-galactosidase and glucocerebrosidase are expected to reach clinical trial phases by the end of 2014 (www.greenovation.com).

The use of *Physcomitrella* as a production host for recombinant biopharmaceuticals was facilitated by well-developed molecular toolboxes. Heterologous as well as endogenous promoters were characterized for their suitability to achieve high levels of recombinant product (Horstmann et al., 2004; Weise et al., 2006). In addition, several moss-derived signal peptides were evaluated for improved secretion of the recombinant product to the surrounding medium (Schaaf et al., 2004, 2005; Weise et al., 2006). The moss genome sequence is available since 2008 (Rensing et al., 2008), and together with nearly 400,000 expressed sequence tags (ESTs) obtained from different experimental conditions, tissues, and developmental stages (Nishiyama et al., 2003; Lang et al., 2008) it allows a reliable prediction of gene structures. The internet resource  provides access to a high-quality functional annotation including more than 32,000 protein-coding genes (Zimmer et al., 2013). This resource was very convenient for the identification of genes involved in the glycosylation of recombinant proteins synthesized in moss bioreactors.

However, the main driver for moss functional genomics approaches in general and glyco-engineering in particular was the unique accessibility of this organism for gene targeting approaches via homologous recombination. Displaying an exceptionally high rate of homologous recombination in mitotic cells (Strepp et al., 1998; Schaefer, 2001; Hohe et al., 2004; Kamisugi et al., 2006), base-specific precise genetic engineering is feasible with high efficiency. Undesirable gene functions can be completely eliminated by targeted knockout approaches. The knockout construct used for the transfection of moss protoplasts regularly consists of 700–1000 bp genomic DNA (homologous regions) flanking each side of a selection cassette, which interrupts or replaces the target gene when indicated (**Figure 1G**). Glyco-engineering of moss was successfully accomplished by various gene targeting approaches (see below).

## PROTEIN GLYCOSYLATION AND MOSS GLYCO-ENGINEERING

Protein glycosylation is a complex and heterogeneous modification which can be classified in two main categories, N- and O-glycosylation. In the former, the carbohydrates are attached to the amide group of asparagine (N) in the consensus sequence N-X-S/T (where X can be any amino acid except proline, and the third amino acid can be either serine or threonine; Mononen and Karjalainen, 1984; Gavel and von Heijne, 1990). *O*-glycans, on the other hand, are attached to the hydroxyl group of serine (S), threonine (T), hydroxylysine or hydroxyproline (Hyp; Varki et al., 2009). In contrast to N-glycosylation, consensus sequences for O-glycosylation in mammals are not well defined or non-existing (Hansen et al., 1998; Julenius et al., 2005).

N-glycosylation in animals is a largely cell-type and species-specific feature (Raju et al., 2000; Croset et al., 2012). Moreover, potential glycosylation sites on a given protein can be either unmodified or occupied by varying glycan structures which result from the maturation of the glycan throughout the endoplasmic reticulum (ER) and the Golgi apparatus (GA), leading to microheterogeneity of glycoproteins (Kolarich et al., 2012). Compared to other higher eukaryotes, plants display more conserved glycan patterns between different species and a less diverse palette of *N*-glycans (Bosch et al., 2013), fascilitating the production of homogeneous glycoproteins.

As higher eukaryotes, plants are able to produce *N*-glycans of the complex type with the core sugar structure Man3GlcNAc2 consisting of two *N*-acetylglucosamine and three mannose residues that is identical to humans (Lerouge et al., 1998; Wilson, 2002). Up to two terminally attached GlcNAc residues are also common between plant and bi-antennary mammalian complex-type glycoprotein oligosaccharides (reviewed in Gomord et al., 2010). Differing from the human structure, which displays a fucose residue 1,6-linked to the proximal GlcNAc moiety, most plant *N*-glycans carry a β1,2 xylose and an α1,3 fucose linked to the glycan core. These sugar structures are common for all land plants analyzed so far, including mosses as the evolutionary oldest group (Koprivova et al., 2003; Viëtor et al., 2003). Their presence raised concerns about plant-produced biopharmaceuticals as they were shown to induce antibody formation in mammals (van Ree et al., 2000; Bardor et al., 2003; Westphal et al., 2003; Bencúrová et al., 2004; Jin et al., 2008). The consequence of an immune response against the pharmaceutical can lead to antibody-mediated reduction of product efficacy as well as to severe clinical complications (Schellekens, 2002).

Consequently, first plant glyco-engineering approaches aimed at targeting the glycosyltransferases responsible for the addition of these two residues. Ten years ago *Arabidopsis thaliana* as well as moss lines lacking β1,2 xylosylation and α1,3 fucosylation have been generated (Koprivova et al., 2004; Strasser et al., 2004). The predominant glycan type of the double knockout moss line for β1,2 xylosyltransferase (XylT) and α1,3 fucosyltransferase (FucT) was the GnGn form (GlcNAc2Man3GlcNAc2; Koprivova et al., 2004). A ΔXylT/FucT genotype is currently in use as genetic background for most of the recombinant products described from moss bioreactors.

Lacking the core fucose, the engineered moss *N*-glycans differ from the human ones which contain an α1,6-linked fucose residue. However, lack of this residue proved to be advantageous for the efficacy of antibodies targeting tumor cells (Shields et al., 2002; Shinkawa et al., 2003; Cox et al., 2006; Schuster et al., 2007). The underlying phenomenon, antibody-dependent cellular cytotoxicity (ADCC), comprises receptor binding and activation of a natural killer cell by an antigen–antibody complex on the target cell surface resulting in lysis of the target cell. Binding and activation of the killer cells was up to 40× more efficient with a monoclonal antibody produced in glyco-engineered, fucose-lacking moss cells compared to the same antibody produced in CHO cells (Schuster et al., 2007).

In addition to the GnGn *N*-glycan form, many plant species display α1,4 fucose, and β1,3 galactose linked to the terminal GlcNAc residues on one or both of the antennae (Wilson, 2001). This trisaccharide Fucα1-4(Galβ1-3)GlcNAc is known as Lewis A (Le^a^) structure. It is synthesized by β1,3 galactosyltransferases (GalT) and α1,4 fucosyltransferases as the last steps of *N*-glycan maturation in the plant GA (reviewed in Gomord et al., 2010). In contrast to the high prevalence of xylose and core fucose residues on plant *N*-glycans, Le^a^ structures are found in a much lower proportion (Fitchette-Lainé et al., 1997; Koprivova et al., 2003; Viëtor et al., 2003; Strasser et al., 2007). However, Le^a^ epitopes were described on recombinant proteins produced in plants (Petruccelli et al., 2006; Weise et al., 2007; Castilho et al., 2013). The production of recombinant human EPO (rhEPO) in both moss and *N. benthamiana*, lead to proteins decorated with high amounts of Le^a^ structures (Weise et al., 2007; Castilho et al., 2013). Although Le^a^ epitopes are found in humans as part of the Lewis-positive histo-blood groups (Henry et al., 1995), they are rarely present in healthy adults, but increased in patients with certain types of cancer (Zhang et al., 1994). Furthermore, antibodies against Le^a^ epitopes are frequent (Wilson et al., 2001). Therefore, it is advisable to remove the respective β1,3 galactose and α1,4 fucose residues from plant-produced recombinant products.

**FIGURE 2:**
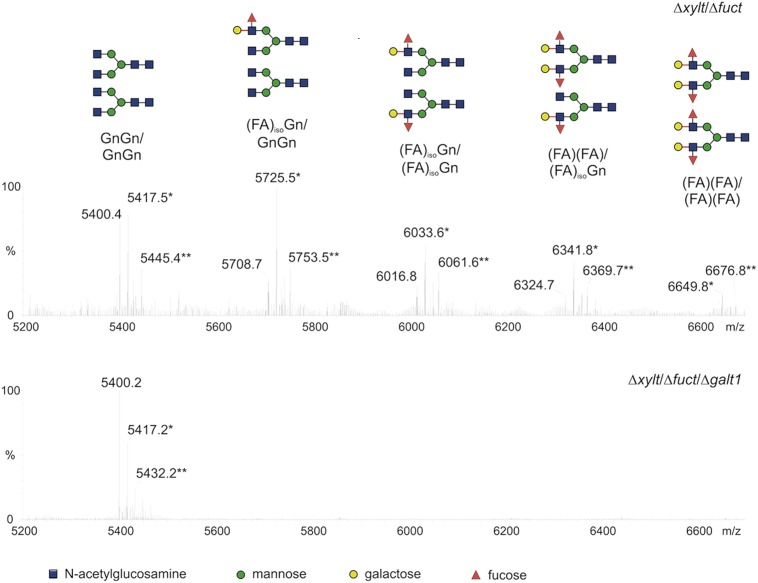
**Glycopeptides of moss-produced rhEPO from two glyco-engineered moss lines.** Comparison of the mass spectra for the rhEPO tryptic peptide harboring two glycosylation sites (EAENITTGCAEHCSLNENITVPDTK) produced in glyco-engineered moss lines: double KO ΔXylT/FucT and triple KO ΔXylT/FucT/GalT (based on Parsons et al., 2012). Salt adducts are marked with asterisks. The glycosylation patterns are schematized with sugar symbols above each peak. Le^a^ structures are totally absent in the triple KO line.

A single putative *α1,4 fucosyltransferase* gene was detected in the moss genome. While the targeted knockout of this gene resulted in the loss of terminal α1,4 fucose residues, β1,3-linked galactoses were still present on moss *N*-glycans. In contrast to the single-copy *α1,4 fucosyltransferase* gene, 13 putative *galt* homologs were identified in *P. patens*. Out of these, exclusively one gene (*galt1*) was shown to be responsible for the synthesis of Le^a^ in moss. The disruption of *galt1* alone resulted in the absence of the complete Le^a^ epitope, not only of the galactose residue but also of the terminal fucose, both in the total moss *N*-glycan pool and on the moss-produced rhEPO (Parsons et al., 2012). The absence of the α1,4 fucose in the *galt1* knockout line (Δ*galt1*) with intact α1,4 fucosyltransferase activity confirmed that this is the last enzyme in the plant N-glycosylation pathway and that the presence of galactose on the substrate is indispensable for the fucosyltransferase activity (Parsons et al., 2012). The lack of GalT1 activity did not affect the moss growth rate. The homogeneity of the rhEPO glycosylation achieved in the moss *galt1* knockout line was remarkable, with almost only one glycosylation form, the aimed core structure with terminal GlcNAc residues (Parsons et al., 2012; **Figure 2**).

In humans, the GnGn glycan is frequently further elongated with galactose added in β1,4 linkage and this is often capped with sialic acid residues. The targeted insertion (“knockin”) of the human *β1,4 *galt** into the moss genome demonstrated the general feasibility of β1,4 galactosylation of moss *N*-glycans (Huether et al., 2005; Parsons et al., 2012). Further terminal elongation of plant *N*-glycans has been demonstrated for *N. benthamiana* which transiently produced glycoproteins with human-like sialylation (Castilho et al., 2013; Jez et al., 2013). This will be a future task for moss glyco-engineering.

Outstanding success has been achieved so far by engineering plant N-glycosylation patterns for the production of humanized glycoproteins. In contrast, the issue of adverse O-glycosylation in PMPs has not been addressed in the same detail. Concerning plant *O*-glycan engineering, recombinant proteins displaying human so-called mucin-type O-glycosylation were generated recently (Daskalova et al., 2010; Castilho et al., 2012; Yang et al., 2012). In contrast to rather conserved N-glycosylation patterns, plant-typical O-glycosylation differs fundamentally from the typical human mucin-type O-glycosylation (reviewed by Gomord et al., 2010), and was shown to induce the formation of antibodies (Léonard et al., 2005). In plants, the main attachment site for O-glycosylation is 4-*trans*-Hyp (reviewed by Showalter, 2001), while no glycosylation of Hyp occurs in animals (Gorres and Raines, 2010). Hyp is generated posttranslationally by prolyl 4-hydroxylases (P4H) via hydroxylation of proline. Prolyl-hydroxylation is a very common modification both in mammals and in plants, though recognition sequences differ. In plants, the target motif for O-glycosylation after P4H-catalyzed hydroxylation, the so-called glycomodules present on Hyp-rich glycoproteins (HRGPs), are defined (Kieliszewski and Lamport, 1994) and validated (Tan et al., 2003; Shimizu et al., 2005). *In silico* analysis of the human proteome revealed that 30% of the human proteins bear a recognition sequence for plant P4Hs (Gomord et al., 2010), thus being putative candidates for non-human prolyl-hydroxylation when recombinantly produced in plant-based systems. In fact, undesired plant-typical prolyl-hydroxylation and in some cases even non-human O-glycosylation of biopharmaceuticals was reported (Karnoup, 2005; Weise et al., 2007; Pinkhasov et al., 2011). The most direct strategy to avoid non-human O-glycosylation in PMPs is the elimination of the anchor Hyp, which itself is an undesired PTM performed by plant P4H enzymes.

After systematic disruption of each of the six *p4h* genes in *Physcomitrella*, targeted deletion of *p4h1* resulted in the complete elimination of the previously reported prolyl-hydroxylation of moss-produced rhEPO (Parsons et al., 2013). As prolyl-hydroxylation and further glycosylation of plant extracellular matrix and cell wall proteins play important roles for growth, cell differentiation, and stress adaption (Lamport et al., 2006; Velasquez et al., 2011) we expected a negative impact on the growth rate of the lines. However, the Δ*p4h1* moss lines were not impaired neither in growth or development nor in protein productivity (Parsons et al., 2013).

The ease of gene targeting in moss enabled glyco-engineering approaches for the elimination of any plant-typical immunogenic residues. This provides a plant-based system offering the stable production of safe protein therapeutics.

## AUTHOR CONTRIBUTIONS

Eva L. Decker, Juliana Parsons, and Ralf Reski were involved in gathering and interpretation of data, writing the manuscript and revising the work. Ralf Reski is co-inventor of the moss bioreactor and co-founder of greenovation Biotech. He currently serves as advisory board member of this company. Eva L. Decker, Juliana Parsons, and Ralf Reski are co-inventors of patents and patent applications related to the topic discussed here.

## Conflict of Interest Statement

The authors declare that the research was conducted in the absence of any commercial or financial relationships that could be construed as a potential conflict of interest.
